# From value orientations to payment: health vs. environmental consciousness and willingness to pay for green hotels via green literacy

**DOI:** 10.3389/fpsyg.2026.1792683

**Published:** 2026-03-16

**Authors:** Zhen Chen, Chengpeng Lin

**Affiliations:** 1 Research Center of China–Pakistan Economic Corridor (Kashi University), Kashgar, China; 2 Department of Business Administration, Chonnam National University, Gwangju, Republic of Korea

**Keywords:** environmental consciousness, green hotel, green literacy, health consciousness, willingness to pay

## Abstract

Growing interest in health protection and environmental sustainability is reshaping hospitality demand, yet it remains unclear how these value orientations translate into consumers’ willingness to pay (WTP) for green hotels. Addressing this value–behaviour conversion problem, we propose a dual-pathway model in which health consciousness (HC) exerts a more direct influence on WTP, whereas the effect of environmental consciousness (EC) depends on consumers’ ability to process and evaluate green-related information. Using survey data from 330 consumers and structural equation modeling, we test the roles of HC, EC, and green literacy (GL). Results show that HC is positively associated with WTP, with GL partially mediating this relationship. In contrast, EC has no direct effect on WTP; its influence operates entirely through GL, suggesting that pro-environmental values may not translate into payment intentions without sufficient sustainability-related knowledge. These findings identify GL as a cognitive conversion mechanism, clarify health-driven versus knowledge-dependent environmental pathways, and offer empirical implications for green tourism development, consumer promotion, and the green transformation of service industries along the China–Pakistan Economic Corridor.

## 1 Introduction

As climate pressure mounts and everyday health concerns become increasingly salient, “going green” in hospitality is no longer a niche moral choice—it is turning into a market test of whether sustainability can be valued, trusted, and ultimately paid for. Green consumption has therefore been positioned as a practical route toward sustainable development in the hotel industry (Lorek and Spangenberg, 2014; Chung, 2020). Against the background of the China-Pakistan Economic Corridor (CPEC), the green transformation and sustainable development of the service industry have become crucial for high-quality regional development, in which the green hotel industry acts as an important representative of tourism and hospitality services. In principle, green hotels—lodging services that combine environmentally responsible operations with health-related service promises—should be well placed to benefit from this shift. Yet the diffusion of green hotels is still constrained by a familiar bottleneck: many consumers express support for sustainability, but their willingness to pay (WTP) a price premium remains insufficient to sustain large-scale adoption (Agarwal and Kasliwal, 2017; Kang and Nicholls, 2021). This gap between “liking” green offerings and “paying” for them is particularly consequential in hospitality, where green investments (e.g., energy systems, materials, certifications, and service redesign) often require a premium to be financially viable.

Consistent with this “demand–payment” tension, industry indicators suggest that sustainable accommodation supply is expanding, while adoption remains uneven across properties and markets. For example, the Global Hotel Alliance’s Green Collection reportedly expanded from 189 properties (March 2023) to 472 properties (2024), reflecting rapid growth in the visibility and branding of “green” hotels, yet also implying that such offerings still represent a curated subset rather than an industry-wide default. In parallel, global consumer surveys continue to show high stated interest in more sustainable travel, reinforcing the notion that attitudinal momentum alone may not guarantee a corresponding willingness to accept price premiums.

Existing studies have examined multiple antecedents of consumers’ WTP for green hotels (Chia-Jung and Pei-Chun, 2014; Agarwal and Kasliwal, 2017; Kang and Nicholls, 2021). Among these drivers, environmental consciousness and health consciousness stand out as two salient value orientations that may shape premium-payment decisions—yet potentially through different psychological routes. The literature, however, has more often treated environmental consciousness as the primary engine of green consumption, while paying comparatively less attention to the distinct role of health consciousness and—more importantly—how these two orientations may translate into WTP through different pathways. In practice, consumers with strong pro-environmental orientations do not necessarily commit economically, a phenomenon widely described as the awareness–behaviour (or attitude–behaviour) gap. This recurring gap suggests that value orientations alone may be insufficient to explain why consumers move from endorsing green ideals to accepting a tangible price premium in the green hotel context.

At the same time, the post-pandemic rise in health consciousness offers an additional and timely lens for understanding green hotel consumption (Sun et al., 2022). Compared with environmental motives—which are often framed as other-oriented and long-term—health motives are typically self-relevant and immediate. When consumers perceive green hotels as delivering cleaner indoor environments, safer materials, or stronger hygiene-related standards, health consciousness may increase WTP via self-care and risk-avoidance considerations. Yet it remains underexamined whether health consciousness mainly complements environmental consciousness, substitutes for it, or operates through an independent route in shaping WTP. Clarifying this issue matters not only for theory but also for practice: if green hotel premiums are driven more by health-related value, then eco-centric messaging alone may underperform; if premiums depend on environmental motives, then the industry must understand when and why these motives translate (or fail to translate) into payment.

To explain why environmental consciousness may not consistently become premium-paying intention, this study focuses on green literacy as a value-to-behaviour conversion mechanism. Green hotel attributes are often information-intensive and difficult for consumers to verify: many sustainability practices are embedded “behind the scenes,” their benefits are multidimensional and partially intangible, and “green” claims can vary widely in specificity and credibility. Under such conditions, consumers’ ability to understand, evaluate, and use green-related information becomes crucial for turning abstract orientations into informed payment decisions. We conceptualize green literacy as a consumer capability to interpret green information, evaluate green practices, and connect them to meaningful outcomes, thereby reducing ambiguity in valuation and potentially narrowing the awareness–behaviour gap in green hotel purchasing contexts.

Informed by the Theory of Planned Behaviour (TPB), Value–Belief–Norm (VBN) theory, and the Health Belief Model (HBM), this study develops and tests a value-to-behaviour framework linking health consciousness and environmental consciousness to WTP for green hotels, with green literacy proposed as a mediating conversion mechanism. In this framing, TPB supports conceptualizing WTP as an intention-like outcome, VBN motivates why pro-environmental orientations may require enabling mechanisms to translate into costly behavioural commitments, and HBM provides a rationale for a more self-relevant risk-reduction route through which health consciousness may translate more directly into premium acceptance. Using survey data from 330 consumers and structural equation modeling, we address the following research questions: (1) Do health consciousness and environmental consciousness exert differentiated effects on WTP for green hotels? (2) Does green literacy mediate the relationships between these value orientations and WTP? By clarifying differentiated influence pathways and the mediating role of green literacy, this study provides empirical implications for green tourism development, green consumption promotion, and the green transformation and sustainable development of the service industry along the China-Pakistan Economic Corridor.

## 2 Theoretical background

### 2.1 Green hotel

The China-Pakistan Economic Corridor (CPEC), as a flagship project of the Belt and Road Initiative, is accelerating the green transformation and sustainable development of the service industry. Tourism and hotel services, as important carriers for the green upgrade of services along the corridor, directly impact the achievement of sustainable development goals within the CPEC framework. The hotel industry, as a core component of tourism services, plays a key role in the “greening” transformation and is a crucial lever for the green upgrade of the service industry. Therefore, gaining a deep understanding of consumers’ willingness to pay for green hotels is of significant importance in promoting green consumption along the corridor, and achieving sustainable development in the service industry under the CPEC framework. In this context, research on global trends in the green transformation of the hotel industry and consumers’ willingness to pay provides important reference for relevant practices within the framework of the China-Pakistan Economic Corridor. Green hotels refer to environmentally friendly lodging facilities that operate on the principles of ecological protection, safety, and public health (Chung, 2020). They promote ecological conservation, the scientific utilisation of natural resources, and the advancement of green consumption. The emergence and growth of green hotels reflect increasing societal emphasis on environmental sustainability and health-enhancing living environments. In their marketing strategies, green hotels integrate ecological values and consumer health considerations, adopt environmentally responsible and resource-efficient management practices, reduce resource consumption across production, operations, and service delivery, minimise waste generation, and ultimately enhance the ecological, social, and economic value of the hotel. Importantly, because green hotel offerings embed both environmental and health-related value propositions, they provide an appropriate context for examining how distinct value orientations (e.g., environmental consciousness versus health consciousness) are translated into consumers’ willingness to pay.

Green hotel marketing is grounded in green marketing principles and is embedded across all aspects of hotel management, operations, and service systems (Chung, 2020). This approach aims to achieve a coordinated balance between organisational interests, consumer needs, and ecological sustainability. By realising full-process greening—from production and service provision to consumption and marketing—green hotels strengthen the functional role of green principles and green marketing. For consumers, green hotel marketing practices better align with their growing demands for healthy, safe, and environmentally responsible services. However, the benefits communicated by green hotels are often multidimensional and partially intangible, which can increase consumers’ information-processing requirements and uncertainty about the credibility or relevance of “green” claims—conditions under which a capability-based construct such as green literacy becomes theoretically salient.

### 2.2 Willingness to pay

Ajzen and Fishbein’s Theory of Reasoned Action (TRA) conceptualises behavioural intention as the immediate antecedent of actual behaviour, reflecting the degree of effort an individual is prepared to exert toward a particular action. In this sense, willingness to pay represents not only a cognitive evaluation of value but also a behavioural intention that converts perceived benefits into monetary commitment. From an economic perspective, willingness to pay refers to the maximum price a consumer is prepared to offer for a product or service, conditional on perceived utility and budget constraints (Hanemann, 1991). As consumption quantity or cost increases, marginal utility typically decreases, leading to a corresponding decline in willingness to pay. Accordingly, willingness to pay in this study is treated as an intention-based construct that captures consumers’ readiness to accept a price premium for green hotel attributes, rather than merely a general positive evaluation.

In the hotel context, consumers’ payment intentions demonstrate a dual-attribute structure shaped by both health-related and environmental considerations. Health consciousness tends to stimulate willingness to pay directly, as consumers exhibit premium acceptance for features such as organic cotton bedding, smart air purification systems, and wellness-enhancing indoor environments. Conversely, environmental attributes often influence payment behaviour through consumers’ ability to recognise, interpret, and evaluate sustainability information, implying that environmental values may not automatically translate into willingness to pay when consumers face information asymmetry or uncertainty about green claims. This distinction helps explain the awareness–behaviour gap: value orientations may increase favourable attitudes, yet premium-payment intentions may remain weak if consumers lack the capability to process green-related information and assess its relevance to their personal outcomes.

Importantly, willingness to pay for green attributes is strongly context-dependent. Consumers of budget hotels typically respond to basic environmental practices—such as waste sorting or water-saving devices—with a limited premium threshold. In contrast, resort hotel consumers, whose stays involve hedonic and experiential motivations, often show higher premium acceptance when provided with more comprehensive sustainability information (Miller, 2023). These differentiated patterns highlight that willingness to pay is shaped not only by attribute perceptions but also by consumption context, value salience, and the extent to which consumers can translate health- and environment-related values into actionable purchase intentions—thereby motivating the inclusion of green literacy as a conversion mechanism in subsequent sections.

### 2.3 Health consciousness

Health consciousness refers to individuals’ awareness, evaluation, and behavioural tendencies related to health information and health-promoting practices (Gould, 1990). It encompasses knowledge of human physiology and health care, recognition of risk factors that influence individual health, attitudes toward healthy lifestyles, and motivation to engage in disease prevention and health-enhancing behaviours. Individuals with high health consciousness tend to adopt behaviours such as balanced nutrition, regular physical activity, smoking cessation, moderate alcohol consumption, and routine medical examinations (Su et al., 2022). Additionally, health consciousness reflects an understanding of the importance of early detection and timely treatment of diseases, as well as proactive engagement in health management. In the green hotel context, health consciousness is especially relevant because consumers may interpret green practices not only as environmental stewardship but also as signals of cleaner indoor environments, safer materials, and stronger hygiene standards; thus, health consciousness can plausibly shape willingness to pay by heightening sensitivity to health-related benefits and risk-reduction cues.

### 2.4 Environmental consciousness

Environmental consciousness refers to an individual’s awareness, understanding, and behavioural tendencies concerning environmental protection (Sanchez and Lafuente, 2010). It reflects the degree to which individuals recognise environmental issues and adopt behaviours that support ecological sustainability. As a philosophical and behavioural construct, environmental consciousness encompasses two core dimensions. The internal dimension captures individuals’ cognitive, emotional, and perceptual understanding of environmental issues, while the external dimension refers to their willingness and commitment to engage in environmentally responsible behaviours. These two dimensions are interrelated and mutually reinforcing, forming the foundation for environmentally conscious decision-making (Khrushch and Karpiuk, 2021). Nevertheless, even individuals with strong environmental consciousness may not consistently exhibit willingness to pay a premium when green attributes are difficult to verify or evaluate, suggesting that the value-to-behaviour conversion process may depend on additional enabling conditions. This observation provides a conceptual basis for introducing green literacy as a mechanism that facilitates the monetisation of environmental values in consumption decisions.

### 2.5 Green literacy

Green literacy is a concept that has evolved from earlier notions such as environmental literacy and ecological literacy, yet it extends beyond knowledge acquisition by emphasising individuals’ capacity to transform ecological values into practical, responsible behaviours (McBride et al., 2013). Jia (2014) frames green literacy as the educational embodiment of ecological civilisation, highlighting individuals’ ability to internalise ecological principles and translate them into everyday practices. Building on this foundation, Kunchamboo et al. (2021) conceptualises green literacy within the broader green development paradigm, defining it as an integrated capability comprising green knowledge, ecological ethical sentiments, green awareness, and pro-environmental behavioural tendencies shaped through learning, cultivation, and environmental exposure. This perspective underscores green literacy not merely as an individual cognitive attribute but as a holistic system involving personal, societal, and ecological interactions. Consistent with this view, green literacy in the present study is positioned as an enabling capability—distinct from value orientations such as health consciousness and environmental consciousness—because it captures consumers’ competence to interpret, evaluate, and apply green-related information in purchase contexts.

Within the hospitality context, green literacy plays a critical role in shaping consumers’ perceptions of green hotels. Green hotels are designed to promote sustainability, safety, and health by adopting resource-efficient operations, reducing waste, and integrating ecological values into marketing, service delivery, and management processes (Lee and Jan, 2019). As sustainable development principles have proliferated, consumers’ cognition of green hotels has expanded from basic environmental practices—such as waste sorting or energy conservation—to a more multidimensional understanding that encompasses environmental management systems, resource circularity, healthy indoor environments, hygiene and safety standards, and broader corporate social responsibility (Salem et al., 2022). Because many of these attributes are credence- or experience-based, consumers often rely on informational cues to judge their authenticity and relevance; therefore, green literacy becomes central to whether such cues are effectively processed and translated into behavioural intentions.

Green literacy influences how consumers evaluate these attributes. Individuals with higher green literacy levels are more capable of identifying, interpreting, and valuing sustainable hotel practices, thereby exhibiting stronger acceptance of green premiums. Moreover, the influence of green literacy is context-dependent: business travellers tend to prioritize health- and comfort-related factors (e.g., air quality, hygiene assurance), whereas leisure tourists are more responsive to ecological friendliness and sustainability-oriented service elements (Carneiro et al., 2024). These differences suggest that green literacy shapes not only consumers’ interpretation of green hotel attributes but also their willingness to translate such cognitions into concrete behavioural intentions, including willingness to pay. Taken together, the above discussion indicates that health consciousness and environmental consciousness provide motivational foundations, whereas green literacy may function as a key conversion mechanism that bridges these value orientations and willingness to pay—thereby motivating the hypothesis developed in the next section.

## 3 Theoretical model and research hypothesis

### 3.1 Theoretical model

YPrior research has shown that consumers’ health consciousness and environmental consciousness are important antecedents of green literacy (Chen et al., 2015). Moreover, health consciousness, environmental consciousness, and green literacy jointly affect consumers’ willingness to pay for green products and services (De Magistris and Gracia, 2008; Yazdanpanah et al., 2015). However, existing studies have predominantly emphasised the direct effects of health consciousness and environmental consciousness on green purchasing behaviours, while paying insufficient attention to the underlying mechanisms through which these effects occur. This limitation is particularly salient in the green hotel context, where many green attributes are credence- or experience-based and consumers must rely on information cues and personal capability to evaluate “how green” (and how health-relevant) the offering truly is.

Although, from a static perspective, both health consciousness and environmental consciousness may positively influence consumers’ willingness to pay, a process perspective suggests that these awareness factors may ultimately shape willingness to pay through green literacy. Consumers with high health consciousness and environmental consciousness may still fail to translate these concerns into payment intentions if they lack the necessary green literacy—i.e., the knowledge base, value internalisation, and behavioural competence required for ecological decision making. In other words, health and environmental consciousness indicate what consumers value, whereas green literacy reflects whether they can effectively interpret and apply green-related information when making monetary trade-offs. Thus, this study posits that consumers’ green literacy acts as a key psychological mechanism linking health consciousness and environmental consciousness to their willingness to pay for green hotels.

**Figure 1:**
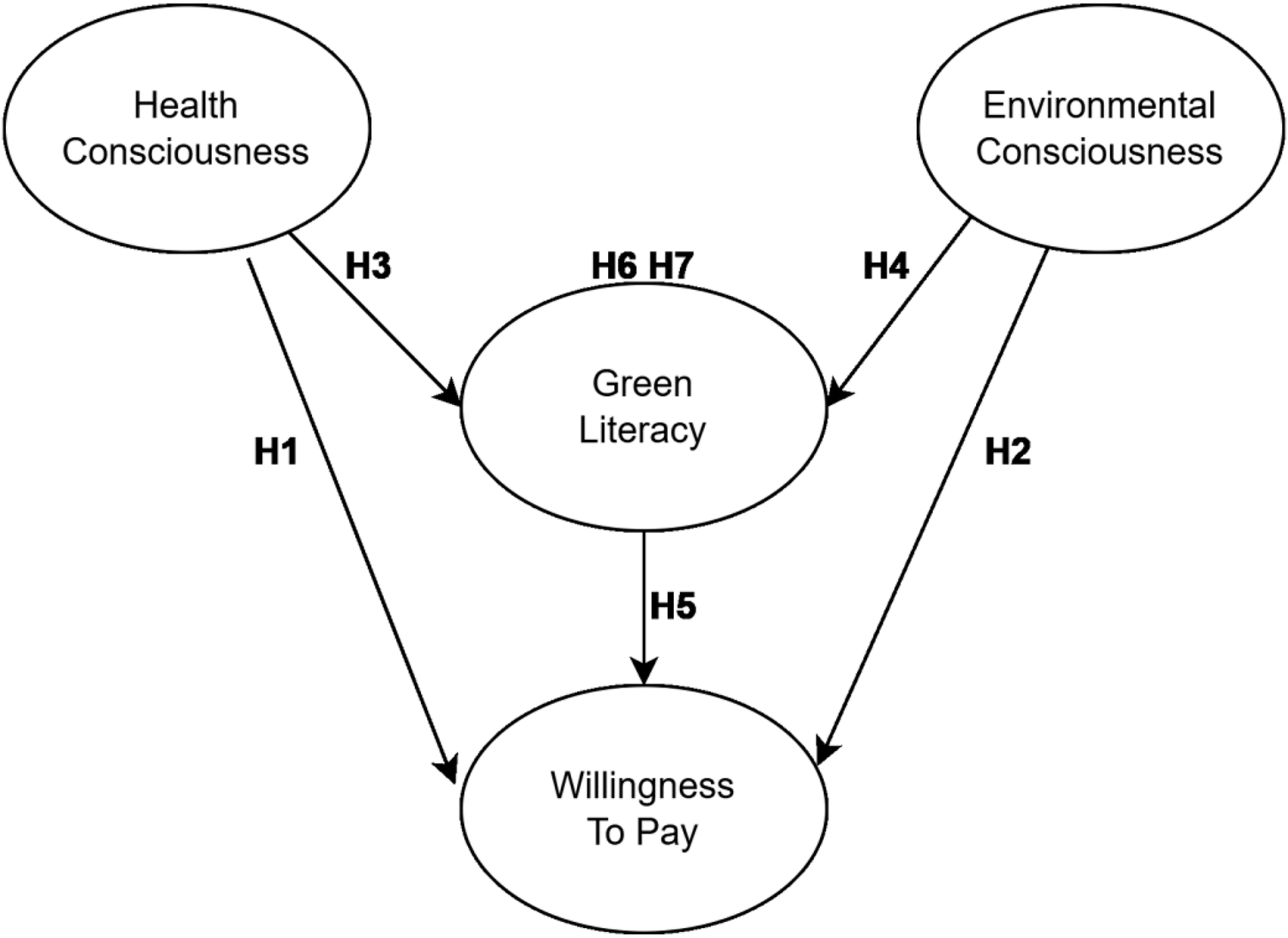
Research model.

Building on this theoretical logic and previous findings, the present study develops the conceptual model illustrated below (Figure 1). The model highlights two primary relationship structures:

First level: Direct effects of health consciousness and environmental consciousness on willingness to pay (H1, H2);

Second level: Indirect effects of health consciousness and environmental consciousness on willingness to pay, mediated by green literacy (H3, H4, H5, H6, H7).

This two-level structure allows the study to examine both (a) whether value orientations exert direct motivational effects on willingness to pay and (b) whether their effects are better explained by a value-to-behaviour conversion mechanism via green literacy.

### 3.2 Research hypothesis

#### 3.2.1 Health consciousness, environmental consciousness and willingness to pay

Consumers’ willingness to pay (WTP) for green hotels reflects a premium-payment decision under perceived benefits and uncertainty. In this context, health consciousness—the extent to which individuals prioritize health and proactively attend to health-related information—can increase WTP because green hotels are often associated with cleaner environments, reduced exposure to harmful substances, and enhanced personal well-being. Prior evidence shows that health-related value orientations are positively associated with premium payment intentions for health-related or green products and services (e.g., Xiong, 2018; Mahmoud et al., 2022; Yang, 2017).

Similarly, environmental consciousness—the extent to which individuals recognise environmental issues and feel concerned about environmental consequences—can motivate consumers to support environmentally responsible offerings through price premiums. Empirical studies in green consumption settings indicate that individuals with stronger environmental consciousness tend to report higher WTP for sustainable products, green packaging, and green hospitality options (e.g., Wu and Huang, 2025; Mahmoud et al., 2022; Yang, 2017). However, some findings also suggest that environmental consciousness may not always translate directly into payment decisions and may operate through more proximal cognitive or responsibility-related mechanisms (e.g., Xiong, 2018).

Taken together, both health- and environment-related value orientations are expected to be positively associated with WTP for green hotels, although the strength and directness of these effects may vary across contexts. Therefore, we propose:

*H1*: Health consciousness positively affects consumers’ willingness to pay for green hotel.

*H2*: Environmental consciousness positively affects consumers’ willingness to pay for green hotels.

#### 3.2.2 The mediating role of green literacy

While value orientations can shape consumers’ preferences, paying a premium for green hotels typically requires consumers to process green-related information (e.g., energy-saving practices, certifications, environmental claims) and to evaluate whether such attributes are credible and personally relevant. Green literacy captures consumers’ ability to identify, interpret, and evaluate green information and to apply such knowledge in consumption judgments and behaviours. In green hotel settings—where “greenwashing” concerns and information asymmetry are common—higher green literacy should reduce uncertainty, strengthen perceived diagnosticity of green claims, and increase consumers’ confidence in justifying price premiums.

Prior research supports the role of value orientations as antecedents of green literacy. Individuals with higher health consciousness are more likely to learn, internalize, and enact green lifestyle knowledge, thereby enhancing green literacy (Li and Zou, 2024; Lin and Niu, 2018). Likewise, environmentally conscious individuals tend to exhibit higher levels of green-related knowledge, attitudes, and behavioural competencies (Goldman et al., 2018; Lin and Niu, 2018). Moreover, green literacy has been shown to facilitate green consumption intentions and behaviours, including willingness to pay for green products (Wei et al., 2018), and to function as a cognitive–behavioural bridge linking upstream drivers to downstream consumption outcomes (Hu and Meng, 2023).

Integrating these arguments, we propose that health consciousness and environmental consciousness enhance willingness to pay partly because they increase consumers’ green literacy, which in turn promotes premium payment intentions.

*H3*: Health consciousness positively influences consumers’ green literacy.

*H4*: Environmental consciousness positively influences consumers’ green literacy.

*H5*: Green literacy positively influences consumers’ willingness to pay for green hotels.

*H6*: Green literacy mediates the relationship between health consciousness and willingness to pay for green hotels.

*H7*: Green literacy mediates the relationship between environmental consciousness and willingness to pay for green hotels.

## 4 Methods

### 4.1 Data sources

Data for this study were collected through China’s largest online survey platform, Wjx.com (Sojump). The survey was administered over a one-month period, from September 1 to September 30, 2025. The survey link was distributed via reputable online part-time recruitment platforms, including Zhubaijie.com and Wike.com, which are commonly used to recruit working adults for academic research in China.

Prior to the main data collection, a pilot test involving 30 respondents was conducted to assess the clarity, comprehensibility, and contextual appropriateness of the questionnaire items. Based on the pilot feedback, minor wording revisions were made to improve readability and ensure contextual relevance, while preserving the original meaning of each scale item.

Because the Wjx.com platform records only fully completed questionnaires, no missing data or item-level nonresponse occurred. A total of 368 completed questionnaires were initially obtained. During the data screening process, 15 questionnaires were excluded due to abnormally short completion times, indicating insufficient engagement, and 23 questionnaires were removed due to patterned or inconsistent responses (e.g., long strings of identical answers or internally inconsistent responses across conceptually related items). As a result, 330 valid responses were retained for subsequent analyses, yielding a valid response rate of 89.67%.

To enhance data quality and reduce potential common method bias, several procedural remedies were implemented. Participation was strictly voluntary, and respondents indicated informed consent by ticking a checkbox before starting the survey. They were informed that they could discontinue participation at any time without penalty. All responses were collected anonymously, and respondents were informed that the survey was conducted solely for academic research purposes. The questionnaire was structured into distinct sections, and clear instructions emphasized that there were no right or wrong answers, thereby reducing evaluation apprehension and socially desirable responding.

The formal questionnaire consisted of three sections: (1) demographic information, including socio-demographic characteristics of the respondents; (2) scales measuring health consciousness and environmental consciousness; and (3) scales measuring green literacy and willingness to pay. All measurement items were adapted from established domestic and international scales related to health consciousness (Su et al., 2022), environmental consciousness (Yang, 2017), green literacy (Goldman et al., 2018), and willingness to pay (Li et al., 2025), with minor contextual modifications to fit the research setting. All variables were assessed using a five-point Likert scale, with response categories ranging from “1 = Strongly disagree” to “5 = Strongly agree”. Original English-language measurement items were translated into Chinese through a rigorous expert-based translation procedure. Specifically, the initial translation was conducted by a bilingual expert with academic training in marketing and consumer behaviour. The translated version was subsequently reviewed and discussed with additional subject-matter experts to evaluate semantic equivalence, cultural appropriateness, and conceptual consistency with the original scales. Based on this expert consultation process, the research team confirmed that the translated items accurately reflected the intended meanings of the original instruments and were suitable for use in the Chinese research context.

### 4.2 Data analysis methods

Because the measurement scales employed in this study have been extensively validated in prior research, confirmatory factor analysis (CFA) was applied to assess the reliability and validity of the constructs. Specifically, CFA was used to evaluate (a) overall measurement model fit, (b) factor loadings, and (c) internal consistency and convergent/discriminant validity of each latent construct before testing the structural paths. Descriptive statistics were first used to examine the frequency distributions, means, and standard deviations of demographic variables and all major constructs. Subsequently, Pearson correlation analysis was conducted to explore the bivariate associations among health consciousness, environmental consciousness, green literacy, and willingness to pay. Partial correlation analysis was additionally performed to control for potential confounding effects of demographic variables. These preliminary analyses served to (a) provide an initial assessment of association patterns and (b) identify potential multicollinearity concerns prior to SEM estimation.

To test the hypothesized relationships among the variables, this study employed structural equation modeling (SEM). SEM was selected due to its methodological advantages: it allows simultaneous estimation of multiple dependent relationships, incorporates measurement error in both independent and dependent variables, enables concurrent assessment of measurement models and structural paths, provides flexibility in specifying latent constructs, and facilitates comprehensive evaluation of overall model fit (Garver and Mentzer, 1999). In line with the proposed mediation logic, the analysis proceeded from the measurement model (CFA) to the structural model (hypothesis testing), enabling direct and indirect effects to be evaluated within a unified framework. Where relevant, demographic variables were included as controls to reduce omitted-variable bias in the estimated structural relations.

## 5 Results

### 5.1 Reliability and validity testing

To ensure the psychometric soundness of all measurement constructs, this study conducted reliability and validity analyses. Internal consistency reliability was assessed using Cronbach’s *α*, and construct validity was examined via the Kaiser–Meyer–Olkin (KMO) measure and Bartlett’s test of sphericity.

The results indicate that all constructs exhibit high internal consistency, with Cronbach’s α values exceeding the recommended threshold of 0.70. The KMO values for all scales were greater than 0.70, and Bartlett’s test reached significance (*p* = 0.000), confirming that the data were appropriate for factor analysis and that the constructs possessed adequate structural validity. Detailed results are presented in Table 1.

**Table 1:** Results of reliability and validity analysis for each variable.

Variable	No. of questions	Cronbach’s *α*	KMO	Sig.
Health consciousness	3	0.832	0.781	0.000
Environmental consciousness	3	0.816	0.763	0.000
Green literacy	4	0.874	0.802	0.000
Willingness to pay	4	0.891	0.827	0.000

### 5.2 Descriptive statistics

A total of 330 valid samples were included in the analysis. Among the respondents, 152 were male (46.061%) and 178 were female (53.939%), demonstrating a relatively balanced gender distribution. In terms of age, 28 respondents (8.485%) were under 18 years old, 187 respondents (56.667%) were aged 18–30, 86 respondents (26.061%) were aged 31–40, and 29 respondents (8.788%) were aged 40 and above. Regarding educational attainment, 67 respondents (20.303%) had an associate degree or below, 198 respondents (60.000%) held a bachelor’s degree, and 65 respondents (19.697%) held a master’s degree or above. Descriptive statistics for demographic variables are provided in Table 2.

**Table 2:** Descriptive analysis.

Variables	Option	Frequency	Percentage (%)	Cumulative percentage (%)
Gender	Male	152	46.061	46.061
	Female	178	53.939	100.000
Age	Under 18	28	8.485	8.485
	18–30	187	56.667	65.152
	31–40	86	26.061	91.212
	Over 40	29	8.788	100.000
Education	High school diploma or below	67	20.303	20.303
	Bachelor’s degree	198	60.000	80.303
	Master’s degree or above	65	19.697	100.000

### 5.3 Correlation analysis

Pearson correlation coefficients were calculated to examine the associations among the study variables. The results revealed significant positive correlations among the core constructs, consistent with theoretical expectations. Health consciousness and environmental consciousness were highly correlated (*r* = 0.751, *p* < 0.01). Green literacy was positively correlated with health consciousness (*r* = 0.807, *p* < 0.01) and environmental consciousness (*r* = 0.820, *p* < 0.01). Willingness to pay was positively correlated with health consciousness (*r* = 0.755, *p* < 0.01), environmental consciousness (*r* = 0.789, *p* < 0.01), and green literacy (*r* = 0.763, *p* < 0.01), supporting the use of structural equation modeling. The complete correlation matrix is reported in Table 3.

**Table 3:** Pearson correlation.

Variables	Gender	Education	HC	EC	GL	WTP
Gender	1.000					
Education	0.032	1.000				
HC	0.087	0.095	1.000			
EC	0.065	0.102	0.751	1.000		
GL	0.043	0.110	0.807	0.820	1.000	
TTP	0.028	0.075	0.755	0.789	0.763	1.000

### 5.4 Measurement model assessment

A confirmatory factor analysis (CFA) was conducted to assess the measurement model. The results indicated an acceptable model fit (*χ*^2^/df = 2.31, RMSEA = 0.064, CFI = 0.952, TLI = 0.903). All standardized factor loadings were significant and exceeded 0.60. Composite reliability (CR) values for all constructs were above the recommended threshold of 0.70, and average variance extracted (AVE) values exceeded 0.50, supporting convergent validity. Discriminant validity was confirmed as the square roots of AVE were greater than the inter-construct correlations (see Table 4).

**Table 4:** Correlation matrix with √AVE.

Variables	CR	AVE	HC	EC	GL	WTP
HC	0.89	0.61	0.87			
EC	0.91	0.64	0.70	0.85		
GL	0.88	0.59	0.69	0.66	0.86	
TTP	0.90	0.66	0.55	0.68	0.59	0.91

Diagonal elements represent the square root of AVE.

### 5.5 Structural equation modeling evaluation and hypothesis

The structural model was estimated using AMOS 21.0. Overall model fit was acceptable (*χ*^2^(98) = 252.116, *p* < 0.001; *χ*^2^/df = 2.551; RMSEA = 0.067; CFI = 0.955; GFI = 0.935; TLI = 0.899), indicating that the model was suitable for hypothesis testing.

With respect to the hypothesized direct effects, health consciousness had a significant positive effect on willingness to pay for green hotels (Estimate = 0.468, *p* < 0.01), supporting H1. In contrast, the direct path from environmental consciousness to willingness to pay was not statistically significant (Estimate = −0.183, n.s.); therefore, H2 was not supported.

Consistent with the proposed mechanism, health consciousness (Estimate = 0.340, *p* < 0.01) and environmental consciousness (Estimate = 0.472, *p* < 0.01) both significantly increased green literacy, supporting H3 and H4, respectively. Green literacy also exerted a strong positive effect on willingness to pay (Estimate = 0.580, *p* < 0.01), supporting H5.

The mediating effects were assessed using bootstrap confidence intervals, with mediation considered significant when the 95% CI excluded zero. The indirect effect of health consciousness on willingness to pay via green literacy was significant (indirect effect = 0.197; 95% CI = [0.111, 0.422]), supporting H6. Because the direct effect of health consciousness on willingness to pay remained significant, this result indicates complementary partial mediation. Similarly, the indirect effect of environmental consciousness on willingness to pay through green literacy was significant (indirect effect = 0.274; 95% CI = [0.033, 0.393]), supporting H7. Given the non-significant direct effect, this pattern is consistent with indirect-only mediation (full mediation).

Overall, the results suggest two pathways to willingness to pay for green hotels: health consciousness influences willingness to pay both directly and indirectly via green literacy, whereas environmental consciousness affects willingness to pay primarily through the indirect route by enhancing green literacy (see Table 5).

**Table 5:** Hypothesis testing results.

Path			Estimate	S.E.	*t*	*p*	Results
HC	→	WTP	0.468	0.102	4.59	<0.001	Supported
EC	→	WTP	−0.183	0.152	−1.20	0.229	Not supported
HC	→	GL	0.340	0.099	3.43	<0.001	Supported
EC	→	GL	0.472	0.111	4.25	<0.001	Supported
GL	→	WTP	0.580	0.098	5.92	<0.001	Supported

Indirect effect(s) of *X* on *Y*				
	Effect	Boot SE	Boot LLCI	Boot ULCI
HC → GL → WTP	0.197	0.079	0.111	0.422
EC → GL → WTP	0.274	0.092	0.033	0.393

## 6 Discussion

This study investigated how health consciousness (HC) and environmental consciousness (EC) re related to consumers’ willingness to pay (WTP) for green hotels by testing a mediation framework centered on green literacy (GL). The findings are consistent with a dual-pathway structure. Health consciousness showed a significant positive direct association with WTP, suggesting that health-related values may be more proximally linked to economic commitment. In contrast, environmental consciousness did not show a significant direct association with WTP, indicating that pro-environmental value orientation alone may not be sufficient to correspond to payment intentions in the green hotel context.

The results also indicate that green literacy is closely associated with the conversion of value orientations into WTP. Specifically, EC was associated with WTP primarily indirectly via GL, consistent with an “indirect-only” mediation pattern in which environmental values are more strongly linked to payment intentions when consumers possess the knowledge and interpretive ability needed to evaluate green hotel practices. This pattern provides a mechanism-oriented account of the often-observed attitude–behaviour gap: consumers may endorse environmental values yet remain reluctant to pay a premium when they lack clarity about what green practices entail, how benefits are produced, or how claims should be assessed.

Green literacy also accounted for part of the association between HC and WTP. This suggests that, in addition to its direct motivational relevance, health consciousness may be linked to higher WTP partly through greater understanding of green attributes and their potential consequences. Taken together, the findings imply that health-oriented motives may relate to WTP both directly and indirectly through cognitive resources that can make green offerings easier to evaluate. Overall, the study highlights that WTP for green hotels is strongest when value orientations co-occur with higher green literacy, which may reduce perceived ambiguity and support more informed valuation.

### 6.1 Theoretical implications

This study makes three theoretical contributions to green consumption and hospitality research.

First, by validating green literacy as a mediator, the study advances mechanism-based explanations for why environmental consciousness often fails to predict green purchasing behaviour directly. Rather than treating the weak EC–behaviour link as inconsistency or measurement noise, the findings suggest that EC can be behaviorally effective when it is converted through a knowledge-based cognitive pathway. This integrates value-oriented accounts of sustainable consumption with competence- and information-processing perspectives, positioning GL as a key construct that reduces uncertainty and improves the evaluability of green attributes in service contexts.

Second, the study clarifies the distinct role of health consciousness in green hospitality decisions. The significant HC → WTP direct path supports the view that self-relevant health values can motivate economic commitment without necessarily requiring extensive cognitive elaboration. Theoretically, this highlights an important distinction between self-oriented motives (health-related) and other-oriented motives (environment-related), suggesting that they may operate through different decisional routes even within the same green consumption setting.

Third, by simultaneously modeling HC, EC, GL, and WTP, the study refines understanding of how multiple value orientations jointly shape green service valuation. The evidence implies that cognitive readiness (green literacy) is a crucial condition for prosocial values to become monetizable intentions, while self-relevant values may exert both direct and indirect effects. This contributes to a more differentiated account of sustainable consumption mechanisms in hospitality markets characterized by information asymmetry and variability in consumers’ ability to interpret green claims.

### 6.2 Strategic and managerial implications

The findings provide empirical support for the green development of the hospitality industry and the sustainable growth of service sector in the context of China-Pakistan Economic Corridor construction.

First, given the robust direct effect of health consciousness on WTP, green hotels should complement eco-centric messaging with clear, customer-relevant communication about health and wellness benefits associated with green features (e.g., reduced exposure to harmful chemicals, improved indoor air quality, and healthier in-room materials). Emphasizing personal health value can activate motivations that translate more readily into premium payment.

Second, because environmental consciousness increases WTP primarily through green literacy, hotels should invest in strategies that build consumer understanding of what their green practices are and why they matter. Practical steps include concise and verifiable sustainability disclosures, easy-to-understand labels, and structured explanations that link specific practices (e.g., energy systems, water conservation, material choices) to tangible outcomes. Such initiatives can reduce ambiguity, improve perceived credibility, and help environmentally concerned consumers translate their values into payment intentions.

Third, the results imply that increasing WTP is not only a matter of strengthening “green attitudes” but also of enhancing consumers’ ability to evaluate green offerings. Accordingly, managers should treat green literacy development as part of the service strategy: designing information cues across the customer journey (booking pages, on-site signage, and post-stay communication) that make green attributes transparent and comparable. Over time, this can support more stable premium acceptance and strengthen the perceived value of green hotel positioning.

## 7 Limitations and future research

This study enriches research on green consumer behaviour in the service industry and offers practical implications for the green transformation and sustainable development of tourism and hospitality services along the China–Pakistan Economic Corridor; nevertheless, several limitations remain and point to promising avenues for future research.

First, the study relied on a cross-sectional design, which constrains causal inference regarding the proposed mediation processes. Although the hypothesized paths are theoretically grounded and the mediation tests are consistent with the data, future research should employ longitudinal, time-lagged, or experimental designs (e.g., manipulating informational exposure to green practices) to more rigorously establish temporal ordering and causal mechanisms linking health consciousness, environmental consciousness, green literacy, and willingness to pay.

Second, all focal constructs were measured using self-reported survey instruments, raising potential concerns about common method bias and social desirability—particularly for environmentally related variables. Subsequent studies could reduce such concerns by adopting multi-source or multi-method approaches, such as combining survey measures with behavioural indicators (e.g., actual booking choices, premium selection, or willingness-to-pay tasks), incorporating objective knowledge tests for green literacy, and using procedural remedies (e.g., temporal separation, marker variables) to strengthen measurement validity.

Third, green literacy was operationalized as a largely linear cognitive mediator. However, the study’s dual-path pattern—where health consciousness shows a direct effect while environmental consciousness operates primarily through green literacy—suggests that consumers may rely on different processing routes when evaluating green hotel attributes. Future work could extend the model by explicitly testing alternative cognitive mechanisms (e.g., perceived diagnosticity of green claims, perceived personal relevance, benefit concretization, or skepticism) and by drawing on dual-process perspectives to examine when consumers engage in heuristic versus elaborative reasoning in green hotel evaluations.

Fourth, the non-significant direct effect of environmental consciousness may indicate meaningful heterogeneity within “environmentally conscious” consumers. Future research should explore segmentation-based explanations (e.g., latent class/profile analysis) to identify subgroups for whom environmental values translate more readily into monetary commitment. Such work could clarify whether the indirect-only pattern reflects information constraints, differences in perceived efficacy, varying thresholds for premium payment, or differential sensitivity to greenwashing risk.

Fifth, the study centered on value-based antecedents and a cognitive bridge (green literacy) but did not incorporate potentially influential market and communication cues that shape how consumers interpret green offerings. Future research could integrate contextual signals—such as certification credibility, transparency of sustainability disclosure, traceability of claims, and the specificity of sustainability storytelling—to examine how external information environments facilitate (or impede) the conversion of values into willingness to pay.

Finally, the generalizability of the findings may be limited by the study’s sample and setting. Replication across countries and hotel segments, as well as comparisons across different types of green services (e.g., eco-resorts vs. urban business hotels), would help establish boundary conditions for the proposed dual-channel framework and assess whether the roles of health consciousness, environmental consciousness, and green literacy vary across institutional and cultural contexts.

Collectively, these directions would extend the proposed framework by strengthening causal evidence, improving measurement rigor, and clarifying when and for whom values translate into willingness to pay through cognitive resources in green hospitality markets.

## Data Availability

The raw data supporting the conclusions of this article will be made available by the authors, without undue reservation.
