# Supplementary material for: From value orientations to payment: health vs. environmental consciousness and willingness to pay for green hotels via green literacy
Source: Front Psychol. 2026 Mar 16;17:1792683. doi: 10.3389/fpsyg.2026.1792683 (PMC13033486; doi:10.3389/fpsyg.2026.1792683)
Supplement: Supplementary file 1 [file Table_1.docx]

Supplementary Material

Figure 1. Research Model


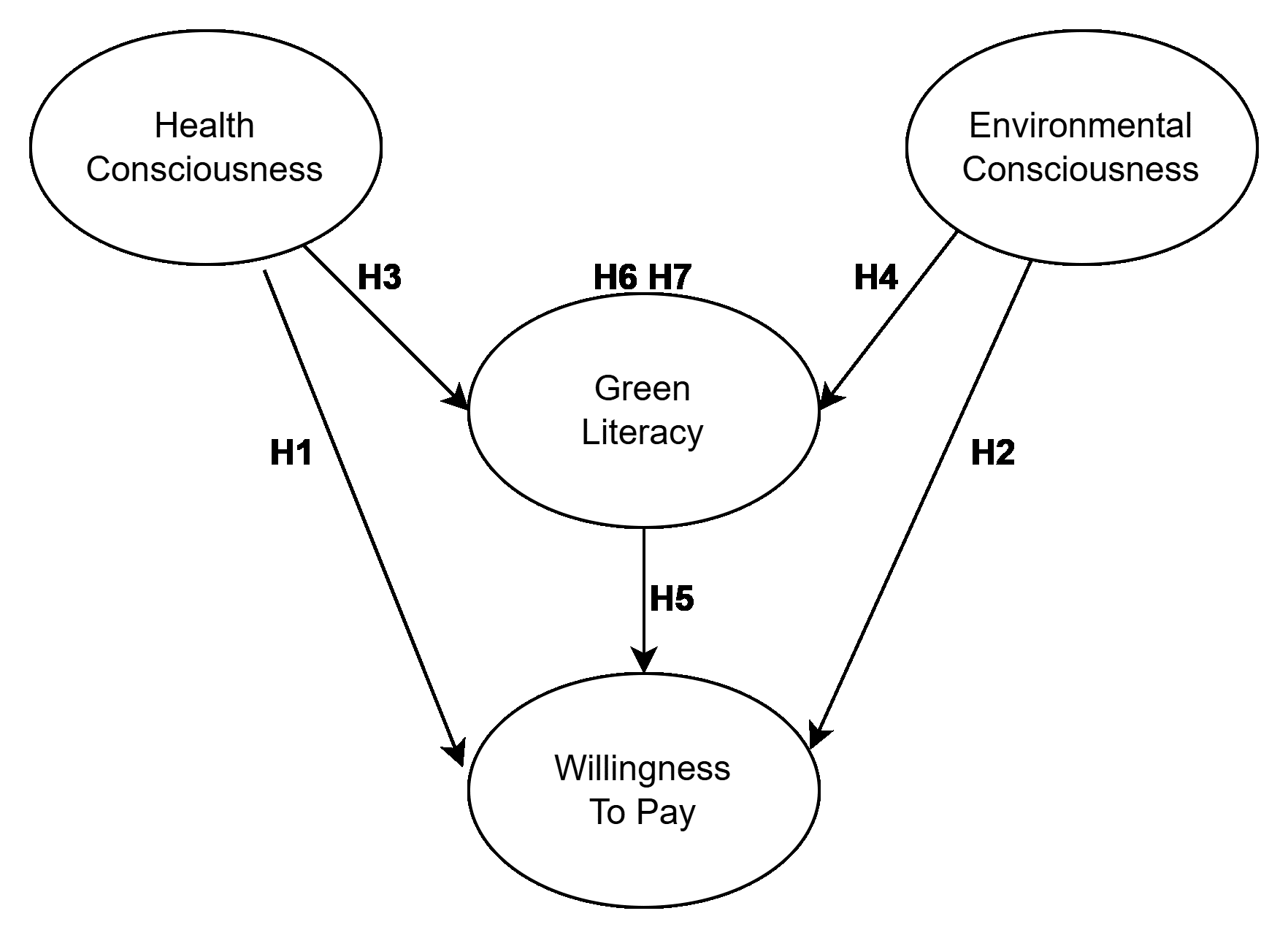


Table 1. Results of reliability and validity analysis for each variable

| Variable | No. of questions | Cronbach’s α | KMO | Sig. |
| --- | --- | --- | --- | --- |
| Health consciousness | 3 | 0.832 | 0.781 | 0.000 |
| Environmental consciousness | 3 | 0.816 | 0.763 | 0.000 |
| Green literacy | 4 | 0.874 | 0.802 | 0.000 |
| Willingness to pay | 4 | 0.891 | 0.827 | 0.000 |

Table 2. **Descriptive analysis**

| Variables | Option | Frequency | Percentage(%) | Cumulative Percentage(%) |
| --- | --- | --- | --- | --- |
| Gender | Male | 152 | 46.061 | 46.061 |
|  | Female | 178 | 53.939 | 100.000 |
| Age | Under 18 | 28 | 8.485 | 8.485 |
|  | 18-30 | 187 | 56.667 | 65.152 |
|  | 31-40 | 86 | 26.061 | 91.212 |
|  | Over 40 | 29 | 8.788 | 100.000 |
| Education | High school diploma or below | 67 | 20.303 | 20.303 |
|  | Bachelor's degree | 198 | 60.000 | 80.303 |
|  | Master's degree or above | 65 | 19.697 | 100.000 |

Table 3. Pearson correlation

| Variables | Gender | Education | HC | EC | GL | WTP |
| --- | --- | --- | --- | --- | --- | --- |
| Gender | 1.000 |  |  |  |  |  |
| Education | 0.032 | 1.000 |  |  |  |  |
| HC | 0.087 | 0.095 | 1.000 |  |  |  |
| EC | 0.065 | 0.102 | 0.751 | 1.000 |  |  |
| GL | 0.043 | 0.110 | 0.807 | 0.820 | 1.000 |  |
| TTP | 0.028 | 0.075 | 0.755 | 0.789 | 0.763 | 1.000 |

Table 4. Correlation Matrix with √AVE

| Variables | CR | AVE | HC | EC | GL | WTP |
| --- | --- | --- | --- | --- | --- | --- |
| HC | 0.89 | 0.61 | 0.87 |  |  |  |
| EC | 0.91 | 0.64 | 0.70 | 0.85 |  |  |
| GL | 0.88 | 0.59 | 0.69 | 0.66 | 0.86 |  |
| TTP | 0.90 | 0.66 | 0.55 | 0.68 | 0.59 | 0.91 |
| Note: Diagonal elements represent the square root of AVE. | | | | | | |

Table 5. Hypothesis Testing Results

| Path | | | Estimate | S.E. | t | p | | Results |
| --- | --- | --- | --- | --- | --- | --- | --- | --- |
| HC | → | WTP | 0.468 | 0.102 | 4.59 | <.001 | | Supported |
| EC | → | WTP | -0.183 | 0.152 | -1.20 | 0.229 | | Not Supported |
| HC | → | GL | 0.340 | 0.099 | 3.43 | <.001 | | Supported |
| EC | → | GL | 0.472 | 0.111 | 4.25 | <.001 | | Supported |
| GL | → | WTP | 0.580 | 0.098 | 5.92 | <.001 | | Supported |
| Indirect effect(s) of X on Y | | | | | | | | |
|  | | | Effect | Boot SE | Boot LLCI | | Boot ULCI | |
| HC → GL →WTP | | | 0.197 | 0.079 | 0.111 | | 0.422 | |
| EC → GL →WTP | | | 0.274 | 0.092 | 0.033 | | 0.393 | |
